# Intramuscular Innervation of the Supraspinatus Muscle Assessed Using Sihler’s Staining: Potential Application in Myofascial Pain Syndrome

**DOI:** 10.3390/toxins14050310

**Published:** 2022-04-28

**Authors:** Hyung-Jin Lee, Ji-Hyun Lee, Kyu-Ho Yi, Hee-Jin Kim

**Affiliations:** 1 Department of Anatomy, Catholic Institute for Applied Anatomy, College of Medicine, The Catholic University of Korea, Seoul 06591, Korea; leehj221@catholic.ac.kr; 2 BK21 FOUR Project, Division in Anatomy and Developmental Biology, Department of Oral Biology, Human Identification Research Institute, Yonsei University College of Dentistry, Seoul 03722, Korea; jh_anatomy@naver.com (J.-H.L.); kyuho90@daum.net (K.-H.Y.); 3 COVID-19 Division, Wonju City Public Health Center, Wonju-Si 26417, Korea; 4 Department of Materials Science & Engineering, College of Engineering, Yonsei University, Seoul 03722, Korea

**Keywords:** supraspinatus muscle, botulinum neurotoxin, shoulder pain, myofascial pain syndrome

## Abstract

Despite the positive effects of botulinum neurotoxin (BoNT) injection into the neural arborized area, there is no anatomical evidence in the literature regarding the neural arborization of the supraspinatus muscle. The present study aimed to define the intramuscular neural arborized pattern of the supraspinatus muscle using the modified Sihler’s staining method to facilitate the establishment of safe and effective injection sites in patients with myofascial pain in the supraspinatus muscle. Seventeen supraspinatus muscles from 15 embalmed cadavers were dissected. Precise suprascapular nerve entry locations were also observed. Intramuscular neural arborization was visualized by Sihler’s staining. The supraspinatus muscle was divided into four portions named A, B, C, and D. The nerve entry points were observed in 88.2% (15 of 17 cases) of section B and 76.5% (13 of 17 cases) of section C of the supraspinatus muscle, respectively. The concentration of intramuscular neural arborization was highest in section B of the supraspinatus muscle, which was the center of the supraspinatus muscle. When the clinician performs a trigger point and a BoNT injection into the supraspinatus muscle, injection within the medial 25–75% of the supraspinatus muscle will lead to optimal results when using small amounts of BoNT and prevent undesirable paralysis.

## 1. Introduction

Shoulder pain is a common musculoskeletal complaint, with a 1-year prevalence range of 20–50%. Shoulder pain is the main contributor to nontraumatic upper limb pain, and chronicity and symptom recurrence are common. In particular, painful or stiff shoulders may be caused by neurological or vascular disorders, myofascial trigger points, and disorders of the cervical vertebrae [1,2,3].

The myofascial tender point (MTP) is a hyperirritable spot in the skeletal muscle that is associated with a hypersensitive palpable nodule. Numerous studies have reported that myofascial pain mainly occurs in the shoulder, neck, and back regions [4,5,6]. A common cause of shoulder pain is myofascial pain caused by MTPs in the rotator cuff muscles. Bron et al. reported a 60% prevalence of myofascial trigger points in the supraspinatus muscle (SS) [7]. Jorge et al. found a similar prevalence of MTPs in the SS in post-stroke patients (68%) [8]. In particular, the MTPs of the SS are moderately correlated with disabilities of the arms, shoulders, and hands [8]. For this reason, it is of paramount importance that symptoms are treated as quickly and effectively as possible, as they may impair one’s quality of life.

Various therapeutic methods have been applied to treat MTPs in the shoulder area, including manual techniques, sprays, stretching, anti-inflammatory drugs, steroids, lidocaine, trigger point injection, and botulinum neurotoxin (BoNT) injections [2,7,9,10,11,12]. The use of BoNT to treat myofascial pain has been investigated in several clinical trials, resulting in significant improvements in pain relief [9,13,14,15,16,17]. Since BoNT hinders the release of acetylcholine at the neuromuscular junction (NMJ), it can cause prolonged muscle relaxation [14]. Consequently, BoNT injections offer a sustained period of pain relief [13,18].

Injecting BoNT to alleviate myofascial pain can be more effective when it is accurately injected into the NMJ of the target muscle [19,20,21], since MTPs are closely related to the densely allocated NMJs [22]. The significance of targeting BoNT injections at the NMJ has been verified through clinical experiments on the biceps brachii and iliopsoas muscles [23,24].

Because the entire human muscle is so large, there is no direct way to determine the location of NMJ distribution. To overcome this, many researchers have used Sihler’s whole mount nerve staining method, which can preserve intramuscular neural pathways [25,26]. Through the modified Sihler’s staining method, it is possible to trace all intramuscular neural pathways. This is safer than the manual dissection method, and it makes it possible to observe the neural arborized area where the fine branches of the nerve are distributed. As a result, it becomes possible to confirm the intramuscular nerve ending (IMNE), which is very close to the densely allocated NMJ. Additionally, neural arborized area-targeted injections result in considerable pain reduction compared to MTP injections [23,24].

Despite the positive effects of BoNT injection into the neural arborized area, there is no anatomical evidence in the literature regarding the neural arborization of the SS.

Therefore, the present study aimed to define the distribution pattern of IMNE of the SS using the modified Sihler’s staining method to facilitate the establishment of safe and effective injection sites in patients with myofascial pain in the SS.

## 2. Materials and Methods

### 2.1. Harvesting Specimens

Seventeen SS (ten on the right side and seven on the left side; eight male and seven female specimens, with a mean age of 80 years) from 15 South Korean embalmed cadavers with no history of trauma or surgical procedures in the scapular region were dissected. We used a total of 15 embalmed cadavers that were provided for use in the student dissection education curriculum. However, some of these cadavers were excluded for reasons such as a partially cut SS, a cut suprascapular nerve that innervates to the SS, or difficulty in confirmation of the origin of the nerve, etc. Therefore, we ended up using 17 sides of the SS from the 15 cadavers that were suitable for the purpose of this study. This study was performed in accordance with the principles outlined in the Declaration of Helsinki. All cadavers were donated to the Surgical Anatomy Education Center of the Yonsei University College of Medicine (approval number: YSAEC 21-005).

The SS was exposed by carefully dissecting the skin, subcutaneous tissue, and trapezius muscle from the scapular region. Meticulous dissection was performed to reveal the suprascapular nerve and artery, which supply the SS. The SS was then removed from the medial border of the scapula between the superior angle of the scapula and the root of the scapular spine to the humeral insertion. After laterally removing the SS, the suprascapular nerve was carefully dissected to identify the nerve entry point to the muscle. The number of nerve fibers entering the muscle and their precise entry locations were observed and recorded. Intramuscular innervation patterns were visualized after performing Sihler’s staining, as described in the Modified Sihler’s Staining section. Following the staining procedure, the SS specimens were equally divided into four sections, each representing 25% of the total length. The samples were observed on a medical film viewer that provided sufficient illumination to reveal the detailed intramuscular course of the nerves. We observed the distribution patterns and localized the dense portions of the IMNE.

### 2.2. Modified Sihler’s Staining

The reagents and the detailed staining protocol used for the modified Sihler’s nerve staining were as follows.

The staining solutions:

− Fixation solution: 10% unneutralized formalin;
− Maceration solution: 3% aqueous potassium hydroxide (KOH) (add 1 mL of 3% hydrogen peroxide to every 1 L of 3% KOH solution for depigmentation);
− Sihler I solution: one volume of glycerin, one volume of glacial acetic acid, and six volumes of 1% aqueous chloral hydrate;
− Sihler II. solution: one volume of Ehrlich’s hematoxylin, one volume of glycerin, and six volumes of 1% aqueous chloral hydrate;
− Neutralization solution: 0.05% lithium carbonate solution;
− Clearing solution: 70–99% formamide.

Overall, Sihler’s staining comprises eight steps:

1. Fixation: The SS specimens underwent fixation for approximately a week in a 10% unneutralized formalin solution.
2. Maceration: The fixed specimens were washed under running water overnight. Then, the SS specimens were macerated and depigmented for 4 weeks in a maceration solution.
3. Decalcification: The macerated SS specimens were placed in Sihler I solution for 48 h for decalcification.
4. Staining: Once the SS specimens were decalcified, they were stained in Sihler II solution for a week. This step stained all tissues, including nerve and muscle fibers.
5. Destaining: The stained SS specimens were again placed in Sihler I solution for approximately 1–3 h. This step destained the muscle fibers, excluding the stained nerves.
6. Neutralization: The destained SS specimens were neutralized under running water for 30 min. They were then placed in 0.05% lithium carbonate for 30 min.
7. Clearing: As a preparatory step for clearing, the SS specimens were cleared with increasing concentrations (70%, 80%, 90%, and 99%) of formamide.

## 3. Results

In every specimen, the suprascapular nerve entered the SS through the scapular notch under the transverse ligament.

### 3.1. Locations of Nerve Entry Points of the SS

**Figure 1:**
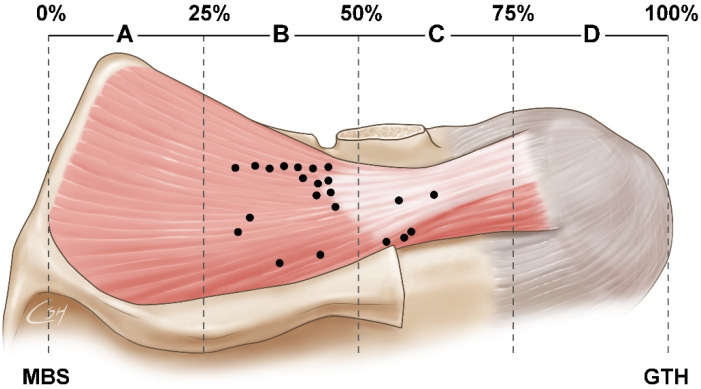
Illustration of the general supraspinatus muscle and the location of the nerve entry points of the supraspinatus muscle. The supraspinatus muscle is equally divided into four portions (A–D) between the medial border of the scapula and the greater tubercle of humerus, each representing 25% of the total length. Black dots indicate suprascapular nerve entry points of the supraspinatus muscle. MBS, medial border of the scapula; GTH, greater tubercle of the humerus.

The nerve entry points were observed in 88.2% (15 of 17 cases) of section B and 76.5% (13 of 17 cases) of section C of the SS, respectively, whereas the other sections (A and D) had no nerve entry points (Figure 1).

### 3.2. Concentration of Nerve Endings in the SS

**Figure 2:**
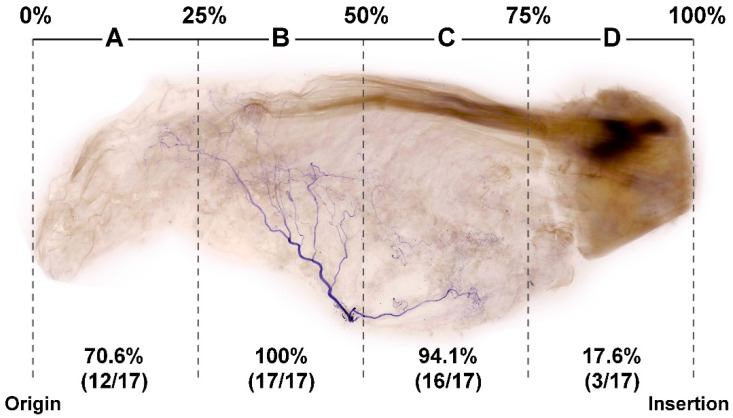

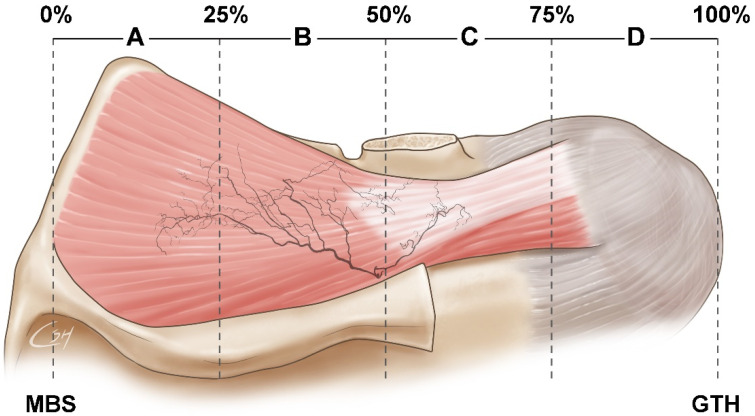
Proportion of intramuscular innervation distribution of the suprascapular nerve to the supraspinatus muscle (**above**), and corresponding illustration (**below**). Violet-stained nerve structures are mostly observed in section B, followed by sections C, A, and D. MBS, medial border of the scapula; GTH, greater tubercle of the humerus.

We tracked the suprascapular nerve course and endings in each specimen. Nerve endings were most frequently observed near the center of the SS (Figure 2). The concentration of the IMNE was highest in section B of the SS (100%, 17 of 17 cases), followed by sections C (94.1%, 16 of 17 cases), A (70.6%, 12 of 17 cases), and D (17.6%, 3 of 17 cases). When the four divided sections were normalized to 100% based on the length from the muscle origin to the point of insertion, the nerve innervated the muscle from approximately the 25–75% region. The intramuscular suprascapular nerve ran medially from the point of insertion to the origin of the SS. Figure 2 illustrates the 25–75% region, which is rich in IMNEs.

## 4. Discussion

The present study aimed to provide an effective BoNT injection site for treating myofascial pain syndrome by clarifying the IMNE distributed in the SS.

The potential MTPs of the SS can be predicted based on the abundance of NMJs [27,28,29]. Travell and Simon [29] and various previous studies [2,7] have suggested tender points of the SS; however, there is no anatomical evidence supporting the potential tender points of the SS. 

In this study, the modified Sihler’s staining method demonstrated the intramuscular nerve course by preserving the NMJs. Most of the neural arborization was observed in sections B and C of the SS, corresponding, respectively, to the 25% to the 75% region of the SS, starting from the medial angle of the scapula (Figure 2). These sections can be found in the mid-region of the SS. This result aligns with the report of Travell and Simon that the MTPs of the SS could be in the mid-region of the supraspinous fossa [29]. Therefore, the location of the region rich in NMJs obtained from this study could be helpful for anatomically predicting the potential MTPs of the SS in clinical settings.

Several studies have revealed that it is likely that MTPs are related to the NMJ areas [30,31,32,33]. Various clinical trials have reported that MTP-targeted injections and BoNT injections resulted in significant pain reduction in patients with myofascial pain syndrome [10,12,23,24]. Xie et al. reported that lidocaine injection therapy with BoNT injection in the NMJ area considerably lessens the intensity and incidence of pain in patients at 6 months following treatment [12]. BoNT relaxes the muscle and reduces muscle pain for an extended period by interfering with the release of acetylcholine at the area of the neuromuscular junction. However, to optimize the effect of BoNT, the injection should be performed near the motor endplate [19]. The effect could be lowered to 50% when the injection is administered 5 mm from the NMJ [34]. Small doses of BoNT result in a reduced treatment effect. In contrast, an overdose of BoNT results in the spread of BoNT to the adjoining muscles, provoking unexpected paralysis. Hence, the accurate localization of the BoNT injection site, based on anatomical information, is essential to achieve a curative effect [27,28].

**Figure 3:**
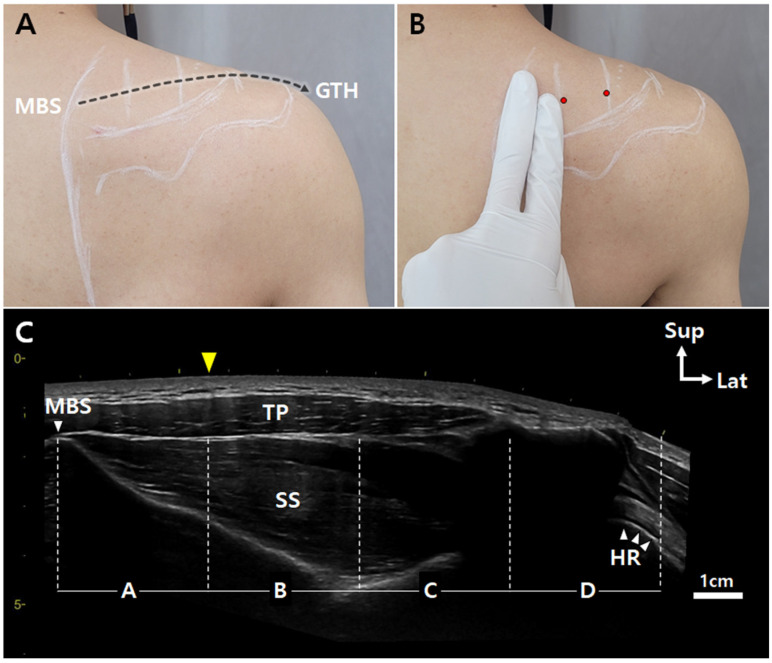
Injection guidelines based on surface landmarks. Ultrasonography scanning was performed from the MBS to GTH (**A**). Location of the injection sites using two fingers from the MBS (**B**). The panoramic view of ultrasonography image showing the four divisions of the SS. The location of 1/4 of the SS can be easily predicted with two fingers (**C**). HR: humerus; TP, trapezius; yellow arrowhead, right next to the second finger; dotted black arrow, ultrasonography scanning site; red circles, injection points; Sup, superior; Lat, lateral.

Physician-friendly surface landmarks can facilitate the localization of MTPs in the SS. Sihler’s staining is the most effective method for observing the intramuscular innervation pathways of skeletal muscles, among several existing research methods that are crucial not only for clinicians, but also for anatomists [35,36]. Therefore, we recommend the following steps to complement and facilitate access to the location rich in NMJs from the skin surface (Figure 3): (1) Palpate the upper medial border and superior angle of the scapula on the skin surface; (2) place two fingers from the medial border of the scapula; (3) define the injection site at 25–75% (Sections B and C); (4) perform the trigger point or the BoNT injection targeting the suprascapular nerve endings. These steps, along with ultrasonography, would enhance the safety and efficacy of the treatment. This surface anatomical landmark allows clinicians to localize the MTP of the SS from the skin surface more easily and ensures a safe and effective BoNT treatment.

One shortcoming of this study is that the specimen shrinks during the staining process when compared with its original muscle morphology, making it difficult to analyze the spatial distribution of the nerve branches to each muscle division. When using Sihler’s staining, the evaluation of the specimen commonly involves a two-dimensional analysis in which individual layers overlap across the entire thickness of the muscle. In contrast, manual dissection techniques can locate where the nerves enter the muscles without the occurrence of spatial shrinkage. Therefore, both techniques were used in this study to clinically apply the anatomical data. Moreover, we suggested a surface anatomical landmark for the application of this anatomical data in a clinical setting. Therefore, the results obtained from this present study would be helpful in managing myofascial pain of the SS using an analgesic agent.

## 5. Conclusions

Currently, there is no standardized injection point for the trigger point or the BoNT injection for the SS. The effectiveness of Sihler’s staining in the SS enables accurate understanding of the IMNE distribution. When a clinician performs a trigger point or a BoNT injection into the SS, injecting 25–75% of the muscle will lead to an optimal result when using small amounts of BoNT, thereby preventing undesirable paralysis.

## Data Availability

Not applicable.

## Abbreviations

MTP	myofascial tender point
BoNT	botulinum neurotoxin
SS	supraspinatus muscle
MBS	medial border of the scapula
GTH	greater tubercle of the humerus
NMJ	neuromuscular junction
IMNE	intramuscular nerve ending
TP	trapezius
HR	humerus
